# Rio’s Mountainous Region (“Região Serrana”) 2011 Landslides: Impact on Public Mental Health System

**DOI:** 10.1371/currents.dis.156b98022b9421098142a4b31879d866

**Published:** 2018-01-25

**Authors:** Marcelo Dell'Aringa, Otavio Ranzani, Joost Bierens, Virginia Murray

**Affiliations:** Division of General Surgery and Trauma, Hospital das Clinicas HCFMUSP, Faculdade de Medicina, Universidade de Sao Paulo, Sao Paulo, SP, Brazil; CRIMEDIM - Research Center in Emergency and Disaster Medicine, Università degli Studi del Piemonte Orientale, Novara, Italy; Universidade de Sao Paulo; Research Group Emergency and Disaster Medicine, Vrije Universiteit Brussel, Brussels, Belgium; Public Health England, London, England; UNISDR Scientific and Technical Advisory Group, Geneva, Switzerland; Integrated Research on Disaster Risk Scientific Committee, Beijing, China

## Abstract

INTRODUCTION In January 2011 landslides and floods followed heavy rain in the Mountainous Region of Rio de Janeiro State (“Região Serrana”), in southeastern Brazil. These events led to the largest disaster registered in Brazilian recent history. Few studies addressed the impacts of this disaster on public health, and we found none addressing the impact on mental health. This study reviewed the consequences of the 2011 disaster in the “Região Serrana”, by comparing the demand for public mental health assistance data from time periods before and after the even  METHODS  We performed an ecologic study, analysing the aggregate data from “Região Serrana” during the period two years before and after the disaster, exporting data from the Brazilian open access public health database. The primary outcome was defined as Mental Health Care Demand, and for that we calculated the number of mental health care visits per month, the proportion of visits due to mental health care and the monthly absolute number of mental health care visits per CAPS – “Centro de Atenção Psicossocial” (Psychosocial Care Centre). For secondary outcomes we evaluated the total number of deaths by any reason, and the total number of hospitalizations. The other health administrative regions of Rio de Janeiro state were used as control group.  RESULTS  We observed that there was an important increase in the rate of visits due to mental health in the six months after the landslides, from 13,875 to 17,690, reaching its maximum one year after the event totalizing 21,980 visits (Dec 2011). It was also observed that the proportion of visits due to mental health disorders increased after the event in the “Região Serrana”, as well as the number of mental health care visits per CAPS.   DISCUSSION  In conclusion, we observed that the 2011 Landslides in “Região Serrana” led to a sustained higher burden to public mental health care. There was an increase in the demand for mental health visits, and the ratio of visits per CAPS was higher during most part of the studied period after the event, even with the region having more CAPS than before.

## Introduction

In January 2011, landslides and floods followed heavy rain in Serrana Region (Região Serrana), the mountainous region of Rio de Janeiro State in southeastern Brasil. This led to the largest natural disaster registered in Brazilian recent history. It claimed the lives of around 918 people and left 30 thousand homeless in 11 municipalities, with seven declaring Estado de Calamidade (Calamity State – on direct translation). A 2014 report counting 845 immediate deaths indicated that 75% were dead by mud burial and 25% by drowning.^1^ The three most affected municipalities were Petrópolis, Teresópolis and Nova Friburgo, with significant immediate damage to the transport and communication systems, as well as to health infrastructure. Furthermore, the local economy suffered with major impacts from the damage to local industry and agriculture; since both urban and rural areas were affected^2^.

Serrana Region is located in the tropical zone, in the central part of the Rio de Janeiro state. It is a mountainous region with altitudes ranging from 400 meters to 2292 meters. The accumulated precipitation of rain in the region can be above 2500 mm in certain municipalities, whereas it is bellow 1300 mm in others^3^. Before the mentioned event, there was accumulated rainfall in order of 241.8 mm in 24 hours, with a peak of 61.8 mm in one hour. The region encompasses 16 municipalities that have medium and high Human Development Index – HDI (0,611 and 0,745)^4^, with a total population of 911,383 inhabitants. It is one of the 9 health administrative regions of Rio de Janeiro State, which is one of the 27 Brazilian federal units. Rio de Janeiro state has an area of 43,780,172 km2 and had a population in 2010 of approximately 16 million inhabitants, while Brazil had 190.755.799 inhabitants(Brazilian Institute of Geography and Statistics - IBGE Census 2010)^5^. See Fig1.

**Map of South America, with Rio de Janeiro State indicated. Zooming in to Rio de Janeiro State map with the municipalities borders depicted by black thin lines, and the 9 health administrative regions highlighted by different colours. Serrana Region is highlighted in red.**
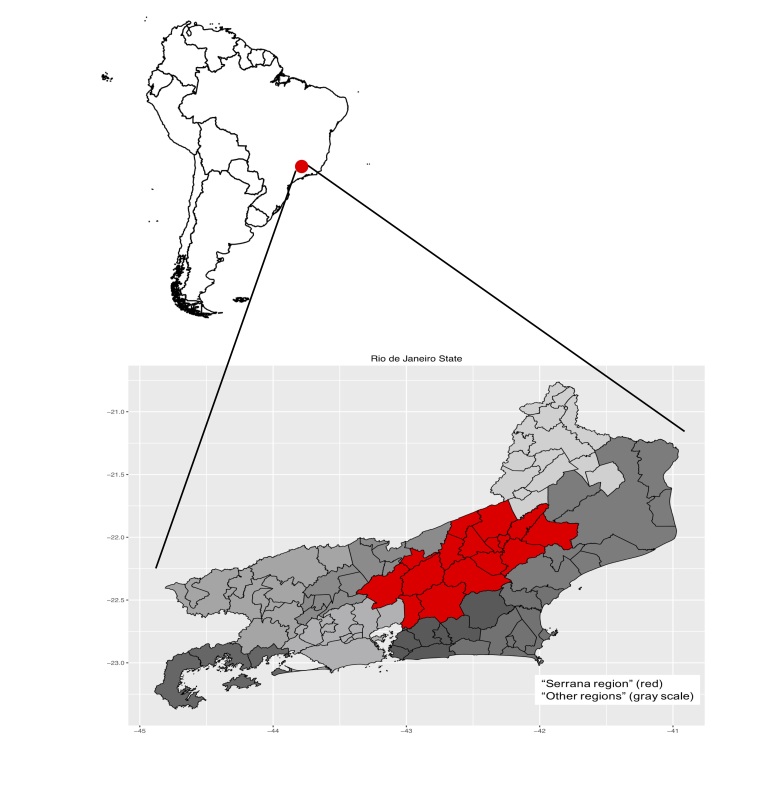

The rainy season in the mountain range region of Rio de Janeiro State (Região Serrana), as in many areas of the south and south eastern regions of Brazil, is during the summer months. Many locations are repeatedly affected by floods and landslides during this period, leading authors to point it as a region under extensive disaster risk^2^ (“risk of low-severity, high-frequency hazardous events and disasters” as suggested by the United Nations terminology). This assumpion is supported by data extracted from the S2iD plataform (Sistema Integrado de Informaçao Sobre Desastres), a Brazilian public access databank on disasters, which registered 241 events in Rio de Janeiro State in 2009 and 2010, leading to 339 deaths and 162.904 people affected^6^ .

Brazil's public health system called Sistema Único de Saúde - "SUS", was created with the constitution of 1988, integrating the legislative goal of universal health coverage. 7 The same constitution gave space for a private supplemental system. A 2013 report by the World bank points out that direct out-of-pocket spending declined over time, but still accounted for around 30 percent of total health spending, while the expenditure on private plans rose and accounted for over 20 percent^8^.****Data extracted from the World Bank open access data-bank show that from the US$1,055.8 per capita Brazil invested in health in 2011, 54,8% came from the private sector. Furthermore, according to IBGE´s open access data from the Research in Medical-Sanitary Assistance 2009, from the 282 psychiatric institutions present in Rio de Janeiro State in 2009, 147 were public (52%), 135 private (47,8%) and 39 public-private mix(13,8%)^9^.

The extensive risk condition leads to direct impact on material losses and potentially high mortality rates along the time. Additionally, the impact of these events may be potentiated through secondary stressors, which are defined in a 2012 systematic review^10^ as “circumstances, events or policies that are indirectly related or non-inherent and consequential’ to the index extreme event” . Some examples are: loss of income, impact on housing value, exposure to negative media reports, physical and mental abuse, loss of social network and lack of access to health and psychosocial care. A report estimates the cost of damage to housing caused by hydrometeorological disasters in Brazil between 2010 and 2014 in R$194 billion (around US$ 95 billion)^11^. Furthermore, two studies evaluated indirect effects of the 2011 disaster in the Serrana Region reporting an important increase in the occurrence of dengue and leptospirosis in the 1-year period following the disaster. 12 ^,^^13^

Most people experience distress after a disaster and less frequently mental disorders can develop following the event. What determines the path to distress that will eventually resolve or to mental illness is not clear, but data suggest it depends on psychosocial resilience and social support^10^. The association between extreme events and mental disorders has been studied in different areas of the world, regarding different types of events, environments and affected populations. For instance, a paper suggested that there was great mental health morbidity after Sarno’s 1998 landslides, in southern Italy. The data suggests that, people in the affected area had higher scores on different domains, like: Anxiety/Sleep Disturbance, subjective efficiency, depression, social impairment and higher incidence of Post-Traumatic Stress Disorder (PTSD)^14^. In a study conducted 33 years after the Aberfan disaster, when a coal slag heap collapse onto a school in Wales, the authors suggested that PTSD was more prevalent between the survivors than in the control group^15^. It is important to notice that the incident still evoked intense feelings and thoughts in many of the survivors who took part of the study. A paper studying psychotic experiences in the population of Sri Lanka, showed that its incidence tends to be higher in those affected by civil conflicts and by the 2004 Tsunami.^16^

Despite all the effort, data on mental illness following landslides are scarce as is shown in a systematic review^17^. The authors discussed that the lack of data may come from the fact that most of the landslides occur in remote regions and poor countries and, frequently landslides come associated to other events such as earthquakes and floods.

To understand an extreme event and its consequences, immediate and long term assessment, taking into account primary and secondary stressors, are beneficial, since the literature shows that the intensity and persistency of both can increase the incidence of mental illness^11^ . This study reviewed the consequences of the 2011 disaster in the Serrana Region, evaluating the demand for mental health care in the SUS, comparing public mental health assistance data from two time periods – two years before and two years after the event. With that we aimed to identify changes on the demand for mental health services provided by the SUS in the affected region.

## METHODS

Study design:

We performed an ecologic study, analysing the aggregate data from Serrana Region during the period before (Jan 2009 to Dec 2010) and after the disaster (Jan 2011 to Dec 2012). We used the aggregate data from the other 8 regions of Rio de Janeiro state as a control group.

Source of data:

We exported data from SUS public access databases using Tabnet, a software developed by DATASUS (Departamento de Informática do SUS, Brazil)18. To evaluate outpatient visits, we assessed the Outpatient Information System (“Sistema de Informações Ambulatoriais – SIA”), the database that contains all data from outpatient visits of the public system. We defined visits due to mental disorders based on which coding the health care workers inputted when the delivered care occurred, the codes are not based on ICD. We defined a priori 35 codes linked with mental illness, excluding those related directly to health problems induced by drugs and alcohol. From the 35 codes, 9 had at least 1 entry, none of them for reasons directly related to drugs and alcohol. All data were retrieved by region of reported address instead of health care facility attendance, because many people may have looked for help or have been referred to other regions of the state.

Mortality and hospital admissions were retrieved from the Mortality Information System (Sistema de Informações sobre Mortalidade – “SIM”) and the Hospitals Information System (“Sistema de Informações Hospitalares do SUS – SIH/SUS”). Demographic data from the Rio de Janeiro state was obtained at IBGE, as the data to build up the region map. The data was exported in May 2016.

Primary outcome: Mental health care demand

We assessed the primary outcome in two additional approaches. In order to correct for possible migration of population, we calculated the proportion of visits due to mental health care (number of mental health care visits / total number of health care visits). In order to correct for possible changes in the availability of health care units, we calculated the monthly absolute number of mental health care visits per each CAPS – “Centro de Atenção Psicossocial” (Psychosocial Care Centre), (total number of mental health care visits / total number of CAPS).

Secondary outcomes:

We evaluated the total number of deaths by any reason, and the total number of hospitalizations. With this we aimed to have a general health impact assessment of the landslides in the studied period and we could also compare the proportion of mental health problems related to the total burden of health problems.

Ethical considerations:

Using ethically agreed principles on open data, it was determined that ethical approval was not needed for this study, since it is anonymised and open to public access on the web.

Statistical plan of analysis:

We reported monthly aggregated data grouped by the affected region (“Serrana Region") and by non-directly affected region (“Other regions”). Data are presented as mean and standard deviation.

We compared the period before disaster occurrence (Jan 2009 to Dec 2010) with the period after the disaster occurrence (Jan 2011 Dec 2012). We calculated the mean difference and its 95% confidence between the periods and used independent t tests to compare both periods.

To analyse the effect of the disaster on our primary outcome, we also performed a quasiexperimental design using interrupted time series (ITS) to control for secular trends in the affected region. The ITS design is a robust design for evaluating the effects of time delimited interventions, allowing adjustments for time trends, estimating changes in baseline levels and account for autocorrelation and seasonal effects19 . We used autoregressive integrated moving average (ARIMA) model, with two parameters to define each segment (before and after the disaster) of our time series. One parameter is the level, which is the value of the series at the beginning of a given time interval. Other parameter is the trend, which is the rate of change of a measure (slope) during a segment. To examine the results, we should analyse whether there are changes in level and trend following an event (i.e, disaster). In general, a change in level constitutes an immediate event effect, and a change in trend represents a gradual variation in the outcome.

We checked the assumptions for the ARIMA model using Phillips-Perron, the Kwiatkowski-Phillips-SchmidtShin and Augmented Dickey-Fuller tests. The autocorrelation was checked by visual inspection of autocorrelograms and partial autocorrelograms of the series and its residuals. The Ljung-Box Q test was run to evaluate a lack of fit of the final ARIMA model.

We considered a P <0.05 to be statistically significant for all of the analyses. The R free source statistical package version 3.2.2 (The R Project for Statistical Computing, Vienna Austria), and the SPSS 21.0 (IBM SPSS, Chicago, IL, USA) were used in all of the analyses.

## RESULTS

We obtained data from the 9 administrative regions of Rio de Janeiro State (Baia da Ilha Grande, Baixada Litorânea, Centro-Sul, Médio Paraíba, Metropolitana I, Metropolitana II, Noroeste, Norte and Serrana) from 48 months, totalizing the expected 4 years period of observation. The data from the not directly affected areas were then grouped (Other regions).

The monthly average number of outpatient visits due to mental disorders was increasing in the affect region before the event, wich is in accordance with data from the Brazilian Ministry of Health that shows an increase in oupatient visits and a decrease in hospitalizations for mental health disorders in Brazil from 2002 to 2014^20^. However, there was a sharp increase in the region in the period after the disaster. Indeed, from Jan 2011 to Jul 2011, there was an important increase in the rate of visits from 13,875 to 17,690, reaching its maximum one year after the event totalizing 21,980 visits (Dec 2011).

In the other regions, there was virtually no change in the average number of visits in the period. We observed a sharp decrease in the last 2 months (November and December 2012) of the time-series from both regions. Fig 2.

**Mental health care visits over time. The green line shows the number of outpatient visits from the not direclty affected area (left y-axis scale) and the brown line shows the number of outpatient visits from the affected area (right y-axis scale):**
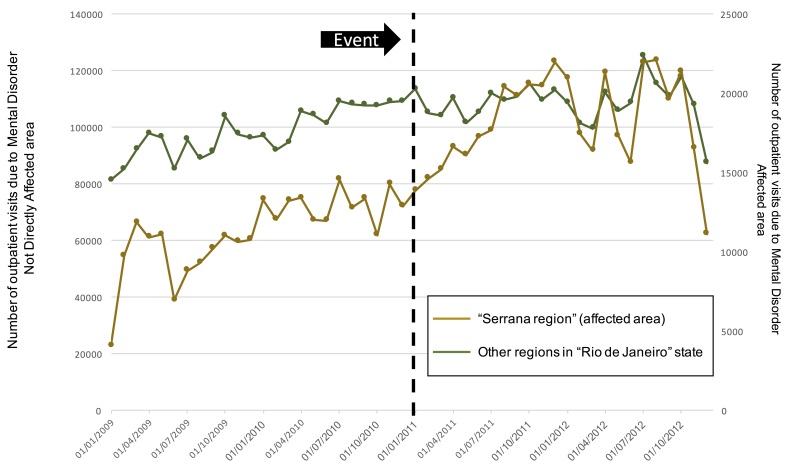

## 

We also evaluated our primary outcome in two different ways aiming to correct by possible changes that could confound our analysis. First, we observed the proportion of the total outpatient visits that was due to Mental Disorders, thus adjusting for possible migration in the affected region. It was observed that the proportion of visits due to mental disorders increased after the event in the Serrana Region, whereas it kept the same level in the other regions of the state (Figure 3-A). In order to adjust for the number of health care units specialized in mental care, we divided the number of mental health visits by the number of mental health units in the region over time. We observed two moments when there was an important increase in mental disorder visits: immediately after the disaster and around 6 months after it (Figure 3-B. Two health care units were installed in the region in the 3 months following the event; this fact probably attenuated the demand in the region.

**Proportion of outpatient visits due to Mental Disorders in Serrana Region The green line shows the number of outpatient visits from the not direclty affected area and the brown line shows the number of outpatient visits from the affected area.**
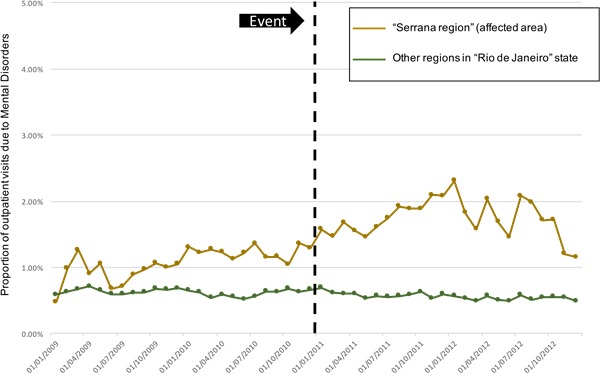

**Number of outpatient visits due to Mental Disorders per Psychosocial Care Centre in Serrana Region and Other Regions over time. The green line shows the number of outpatient visits from the not direclty affected area and the brown line shows the number of outpatient visits from the affected area.**
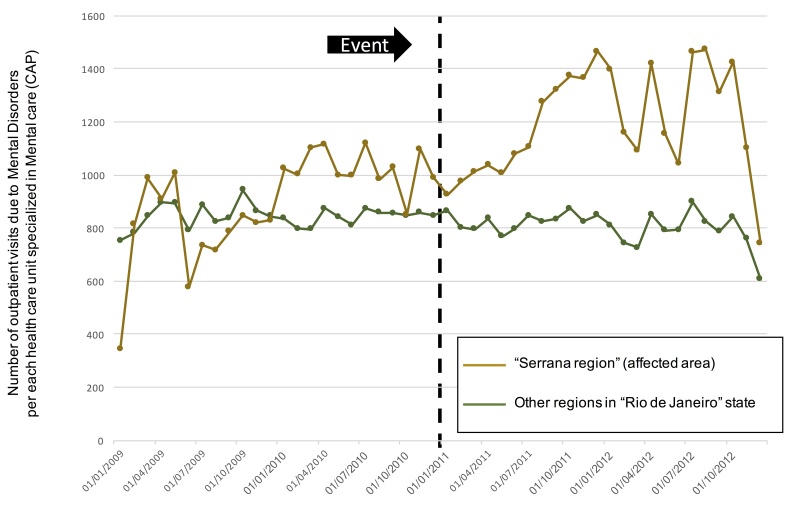

There was an underlying natural trend for higher mental health care visits in the affected region before the event (Coefficient 196.5, p=0.005), and we identified a change in the level of the time series immediatly after the landslide period (Coefficient 3293.1, p=0.017). We could not observe a statistically significant change in the slope of the time series after the event (Coefficient -63.8, p=0.550) (Figure 4).

**Interrupted times series analysis for the primary outcome.**
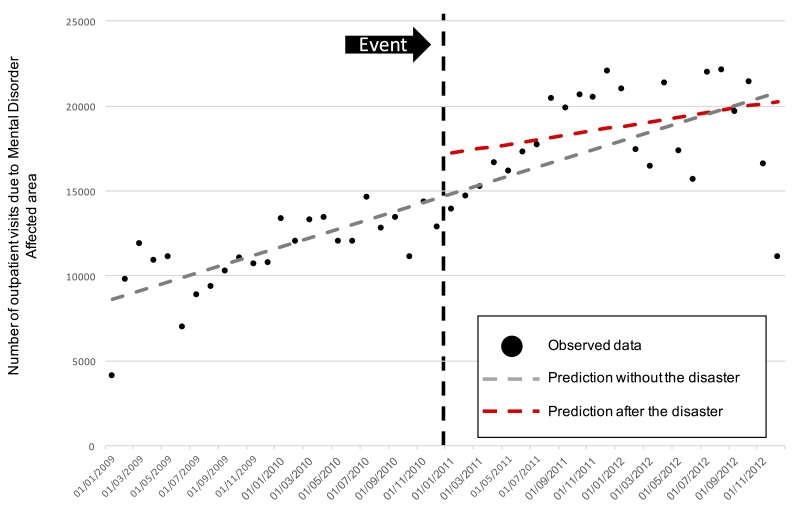

We did not observe any change associated with hospitalization by any reason. Nevertheless, we observed an important peak on the number of deaths immediately after the landslides occurrence (Figures 5 and 6).

**Hospitalizations due to any reason in the Rio de Janeiro state aggregated in the affected and not affected areas over the 4 years period The green line shows the number of outpatient visits from the not direclty affected area (left y-axis scale) and the brown line shows the number of outpatient visits from the affected area (right y-axis scale).**
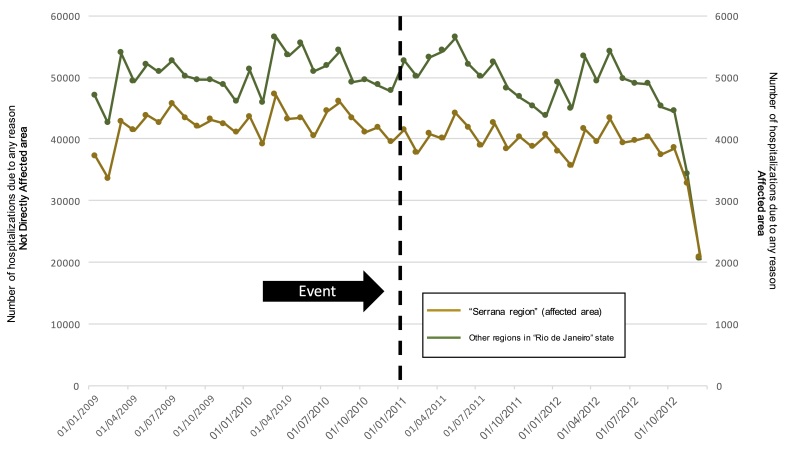

**Number of deaths due to any cause in the Rio de Janeiro state aggregated in the affected and not affected areas over the 4 years period The green line shows the number of outpatient visits from the not direclty affected area (left y-axis scale) and the brown line shows the number of outpatient visits from the affected area (right y-axis scale).**
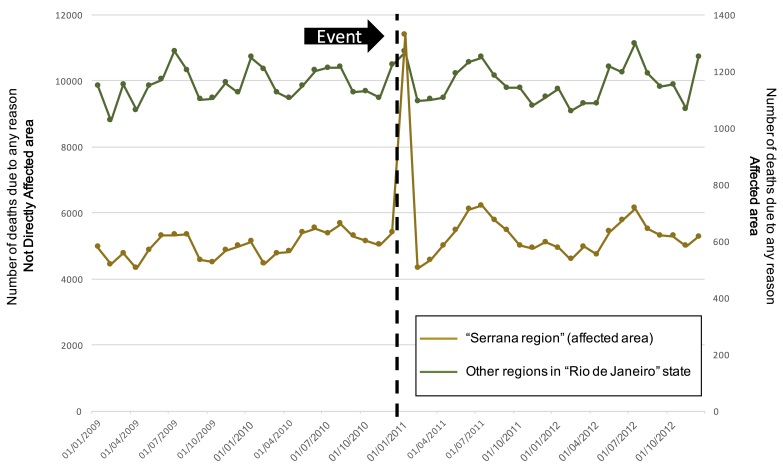

**Table 1:** Average impact of the disaster in the “Serrana region”

	Before	After	Mean difference (95% CI)	P value
Primary outcomes	Mean(standard deviation)	Mean(standard deviation)		
Monthly average number of outpatient visits due to Mental Disorders, n	11,262 (2358)	18,183 (2980)	6921 (5360 to 8482)	<0.001
Monthly average proportion of outpatient visits due to Mental Disorders (Mental disorders / Any reason), %	1.08% (0.2)	1.74% (0.3)	0.67% (0.51 to 0.81)	<0.001
Monthly average number of outpatient visits due to Mental Disorders per each health care unit specialized in Mental Care per month, rate (n/centre)	904 (185)	1197 (203)	293 (181 to 406)	<0.001
Number of health care units specialized inMental Care (“CAPS”)	13 (1)	15 (1)	3 (2 to 3)	<0.001
Secondary outcomes				
Monthly average number of outpatient visits due to any reason	1,046,258 (77971)	1,051,011 (106,174)	4,752 (-49,372 to 58,877)	0.860
Monthly average number of hospitalizations due to any reason	4218 (299)	3884 (457)	-332 (-554 to -110)	0.004
Monthly average number of hospitalizations due to emergencies	2851 (214)	2787 (353)	-64 (-234 to 105)	0.449
Population estimated	920,724 (9542)	919,690 (2764)	-1034 (-5116 to 3048)	0.613

**Table 2:** Average impact of the disaster in the not affected region

	Before	After	Mean difference (95% CI)	P value
Primary outcome	Mean(standard deviation)	Mean(standard deviation)		
Monthly average number of outpatient visits due to Mental Disorders, n	98,237 (8409)	108,792 (7286)	10,555(5984 to 15127)	<0.001
Monthly average proportion of outpatient visits due to Mental Disorders (Mental disorders / Any reason), %	0.63% (0.1)	0.56% (0.1)	0.06% (-0.09 to -0.04)	<0.001
Monthly average number of outpatient visits due to Mental Disorders per each health care unit specialized in Mental Care per month, rate (n/centre)	844 (42)	806 (59)	-38(-67 to -8)	0.015
Number of health care units specialized in MentalCare (“CAPS”)	116 (8)	135 (4)	19 (15 to 22)	<0.001
Secondary outcomes				
Monthly average number of outpatient visits due to any reason	15,819,301 (1,871,855)	19,456,431 (1,567,391)	3,637,130 (2,633,996 to 4,640,264)	<0.001
Monthly average number of hospitalizations due to any reason	50,293 (3239)	47,854 (7464)	-2440 (-5783 to 904)	0.149
Monthly average number of hospitalizations due to emergencies	36,828 (2198)	34,922 (5434)	-1906 (-4314 to 502)	0.118
Population estimated	15,079,434 (907)	15,252,332 (57,856)	172,898 (149123 to 196673)	<0.001

## DISCUSSION

We observed an increase in the demand for public mental health care after the landslides in Serrana Region in 2011, which was not followed by the unaffected area of the state, used as a control. The number of mental health visits remained above the predicted for an almost two-year period after the event. Furthermore, the number of mental health visits per CAPS increased and remained above the baseline for most of the studied period after the landslides. These data indicate that it is likely that the mental health units were busier than previously. We hypothesize that, if surge capacity strategies were not discussed beforehand, the population may have had insufficient support, with worsening of the provided services. This could have contributed, as a secondary stressor, to the impact on the affected population´s mental health.

It is important to notice that the World Health Organization (WHO)^21^ states that “Landslides cause high mortality and few injuries” and that is supported by data from the Landslides in Chuuk 2002, when 90% of the decedents died immediately 22. As well as by the data on the 2014 report by Pereira et al, where they estimate that 136 patients were triaged yellow or red in the hospitals of Serrana Region in the first 24 hours after the landslides, whereas 845 people were considered immediately dead^1^ . In our study, it is evidenced by a sharp peak in mortality rate right after the event.

This study adds to a field where there is paucity of data - mental health consequences of landslides. In the 2015 Systematic Review of Heath Impacts of Landslides^17^ they categorized the included studies on mental health impacts in three categories: two studies on psychosocial support and the moderating effects of family roles, six on the prevalence of psychiatric disorders and one addressing the needs of firefighters who intervened on a landslide. They do not mention any study addressing specifically public mental health assistance impacts. However, the World Health Organization (WHO), has mental health assistance as a major concern after landslides, as mentioned on their News after the 2014 Badakhshan landslide on north-eastern Afghanistan^23^ .

Many of the trends in global disaster risk expressed on the United Nations’ 2015 Global Assesment Report on Disaster Risk Reduction (GAR2015)^24^ are present in our analysis of the herementioned phenomenom: extensive risk, underestimation of risk, indirect disaster losses and impact on future development of underdeveloped countries. Previous analyses and data support the idea that the 2011 Disaster in Serrana Region was linked to important social vulnerability and its consequences should not be addressed merely as a product of chance. For instance, the Pan American Health Organization (PAHO) estimates that 73% of the population and 67% of the health facilities in Latin America and the Caribbean were located in risk zones in 2012^11^. In a 2011 analysis from the Brazilian Ministry of Health in four of the affected municipalities by the 2011 Landslides, 81% of the health facilities were in risk zones^2^. Data from the Brazilian Ministry of the Environment shows that 92% of the 657 landslides recorded during the 2011 event occurred in areas where the vegetation was not well preserved, and they suggest that part of the damage could be minimized if there weren´t people living less than 30 metres away from rivers and in the steeper portions of the region.^25^

Moreover, we see this paper as relevant, since few studies addressed the impacts of the 2011 event in Serrana Region, and many localities in southern and southeastern Brazil are likewise under extensive risk of flood and landslides. Therefore, evaluating the impact of these events is of great importance, since part of the lowered perception of risk could be linked to the lack of long term interventions and secondary stressors identification, issues that we try to analyse in this paper. In conclusion, our study is an effort to address what the Sendai Framework considers a priority: Understanding disaster risk. We observed that the 2011 Landslides in Serrana Region led to a higher burden to public mental health care and that this event is part os a series of events that have been happening in the region as in other areas of the country for a long time.

Recommendation: Recognising this study has shown significant impacts in time and space on mental health, it may be helpful to consider using similar methodologies to repeat this work to see if it is a consistent finding from other landslides, extreme weather events or other incidents. It would also be important to design studies to identify vulnerable populations, and evaluate the difference in mental health access between the population with access to private health services and those that rely exclusively on SUS.

## LIMITATIONS

Our study has important limitations. One limitation of our study is the fact that we collected the data retrospectively and part of the input of data was during and following a disaster, which could impact the accuracy of reported data. Another limitation we need to point out is that we only used data from the public health system and, in Brazil, the private sector accounts for an important part of health assistance. We should also point out that the increase in demand for mental health care may be overestimated because people may have become more aware of mental illnesses in the aftermath. The codes of mental health related procedures were chosen by us from a list with all the possible health procedures from Brazilian Public Health System (SUS), since there is not a pre-defined way to search for mental health related interventions. We did not intend to study drug and alcohol related mental illness, nevertheless one code is related to it, but it did not have any entries. Finally, we found barriers to obtain data for the analysis of mental health care. For instance, we preplanned to analyse the consumption of psychotropic drugs in the region. However, we could not gather this information in the region, even though in Brazil all the distribution of this kind of medication is registered. There is data on the consumption per federal unit that is of easy public access, but we could not find it per health region or municipality.

## Data Availability

The data underlying this study are full available online from the Federal Governmental Secretary, through the Data Mortality Information System (SIM) and the Hospital Information System (SIH/SUS) data available at the DATASUS website (www.datasus.gov.br).

The main data used for this analysis are available from the figshare repository at the following DOI: 10.6084/m9.figshare.5455132

## Competing Interests

Professor Virginia Murray serves on the editorial board of PLOS Currents Disasters. She has not influenced or played any role in the peer-review, editorial decision making or publication of the manuscript.

This paper is the result of a thesis submitted in partial fulfilment of the requirements for the degree of Master of Science in Disaster Medicine (European Master in Disaster Medicine - EMDM).

## Corresponding Author

Marcelo Farah Dell'Aringa: mardellaringa@gmail.com
